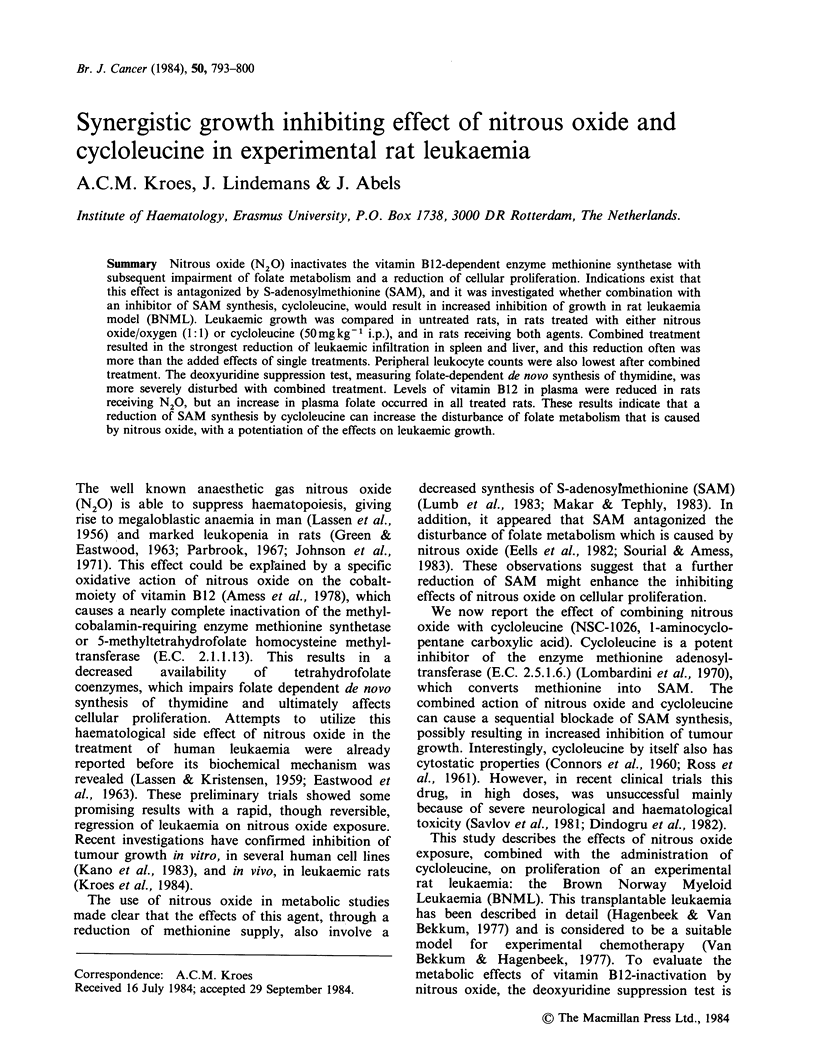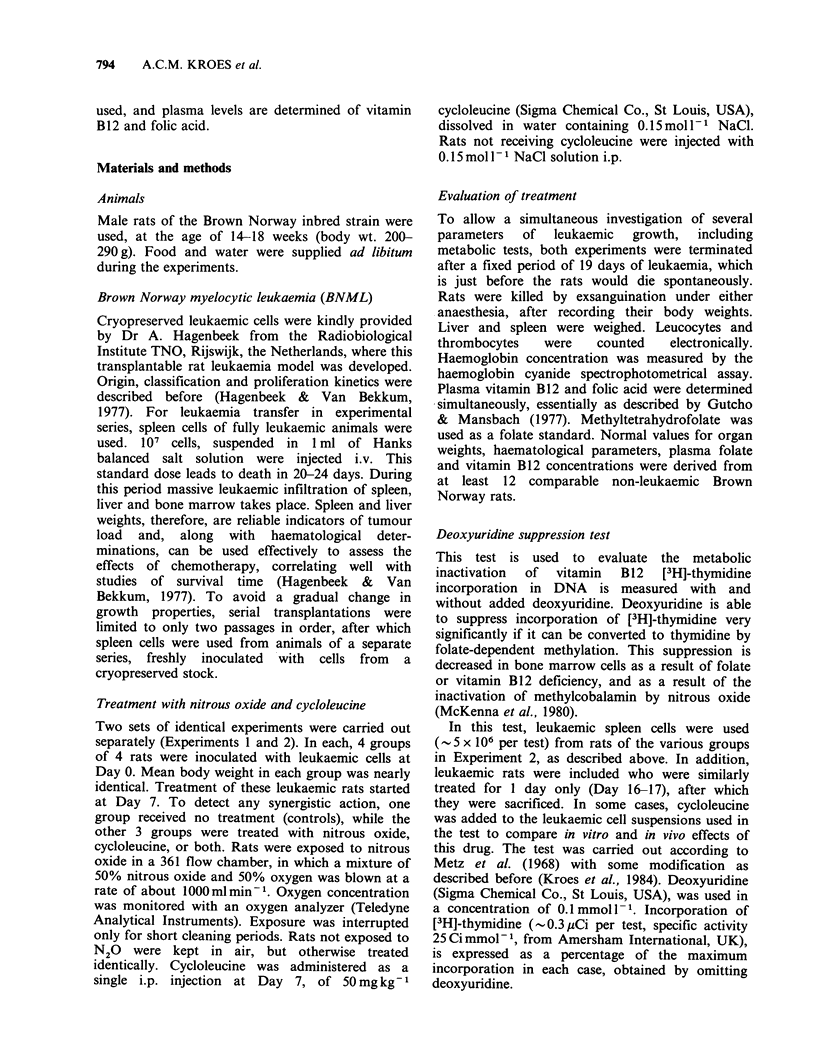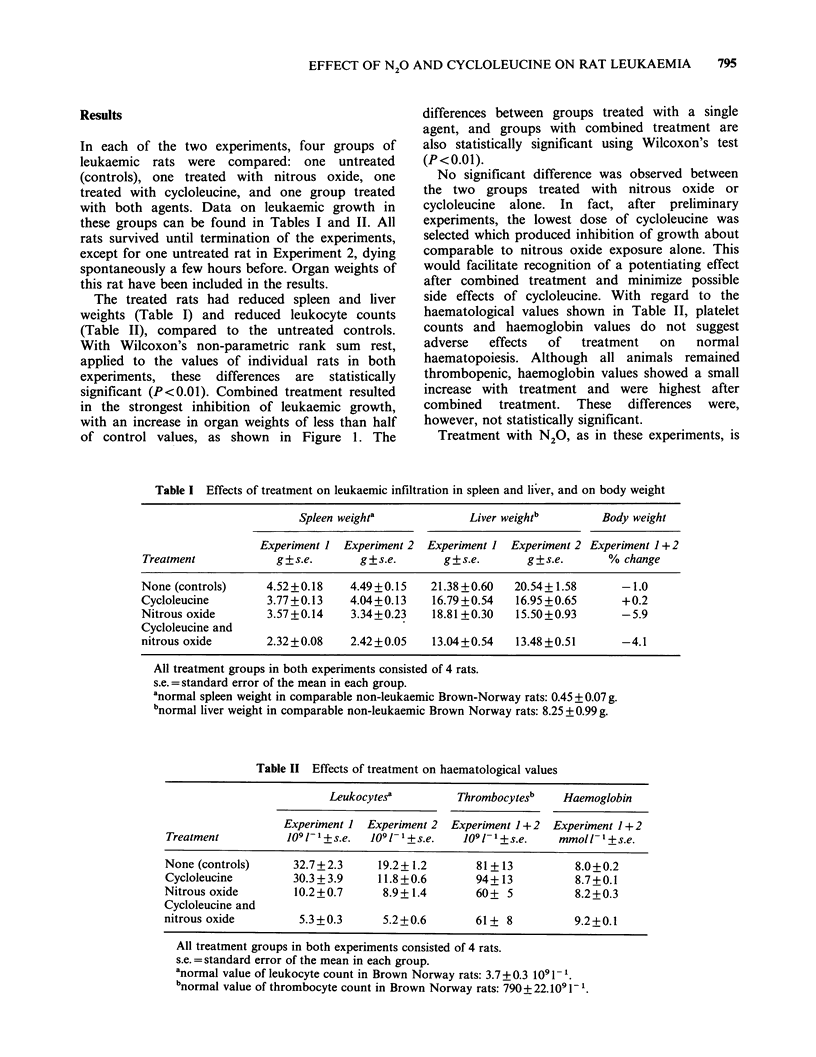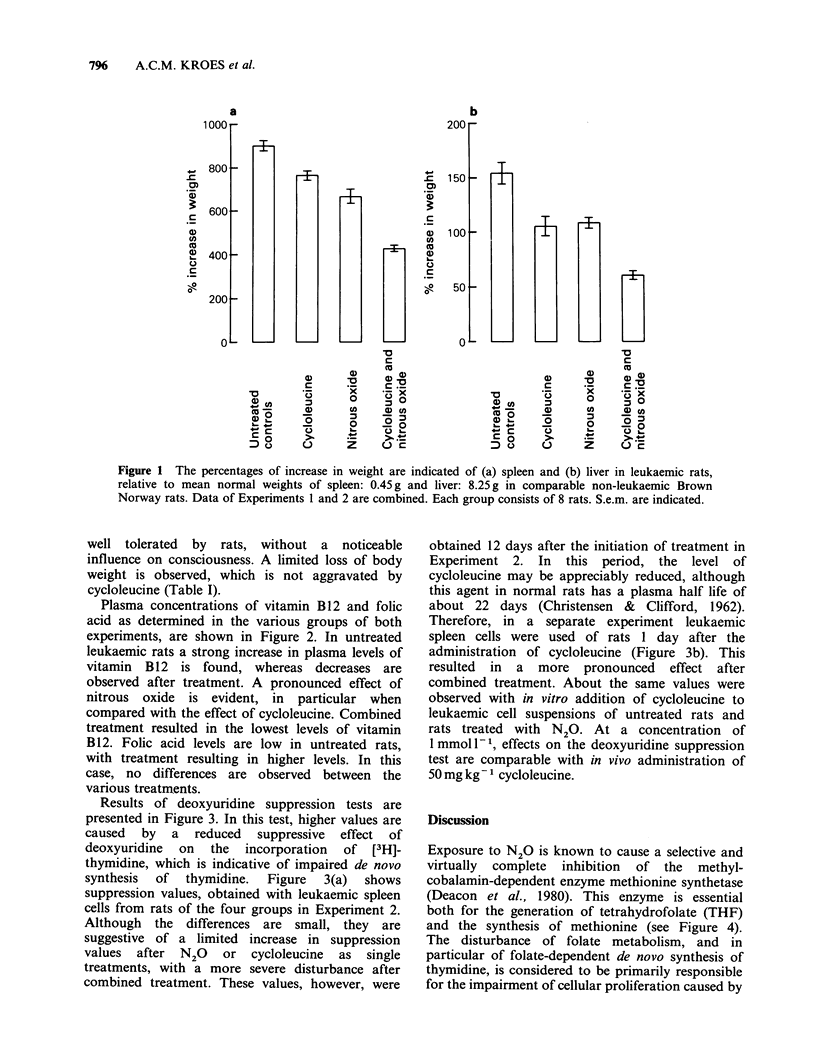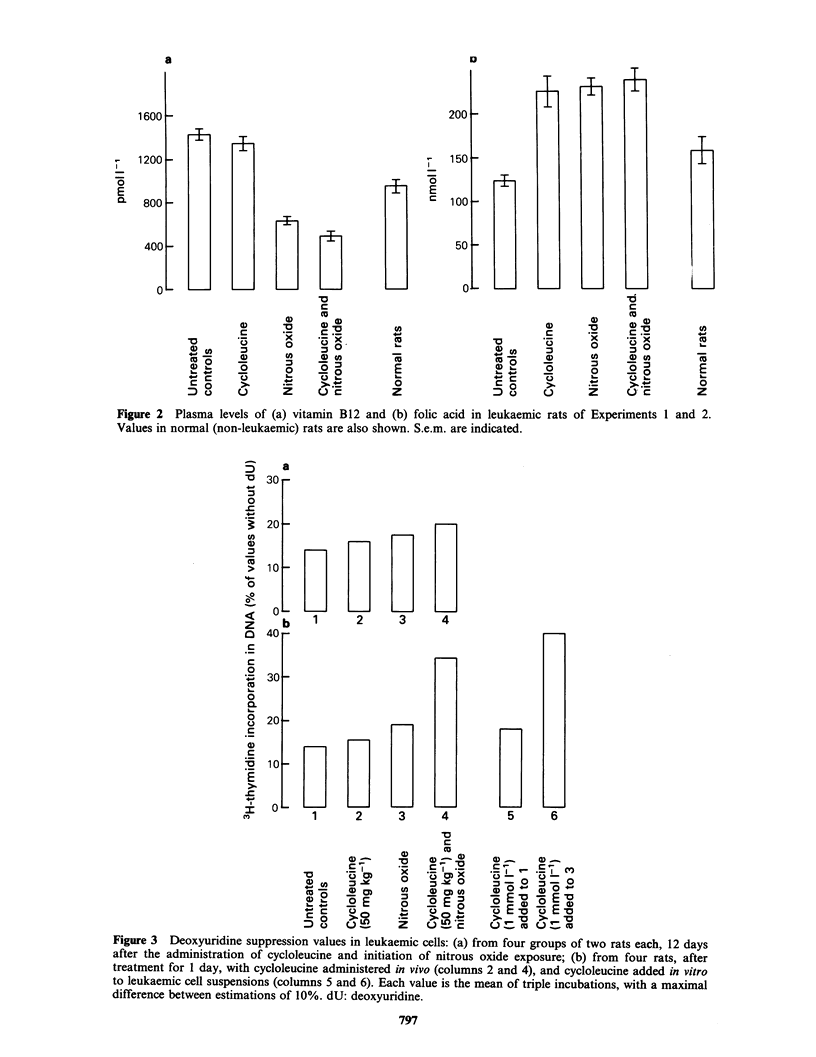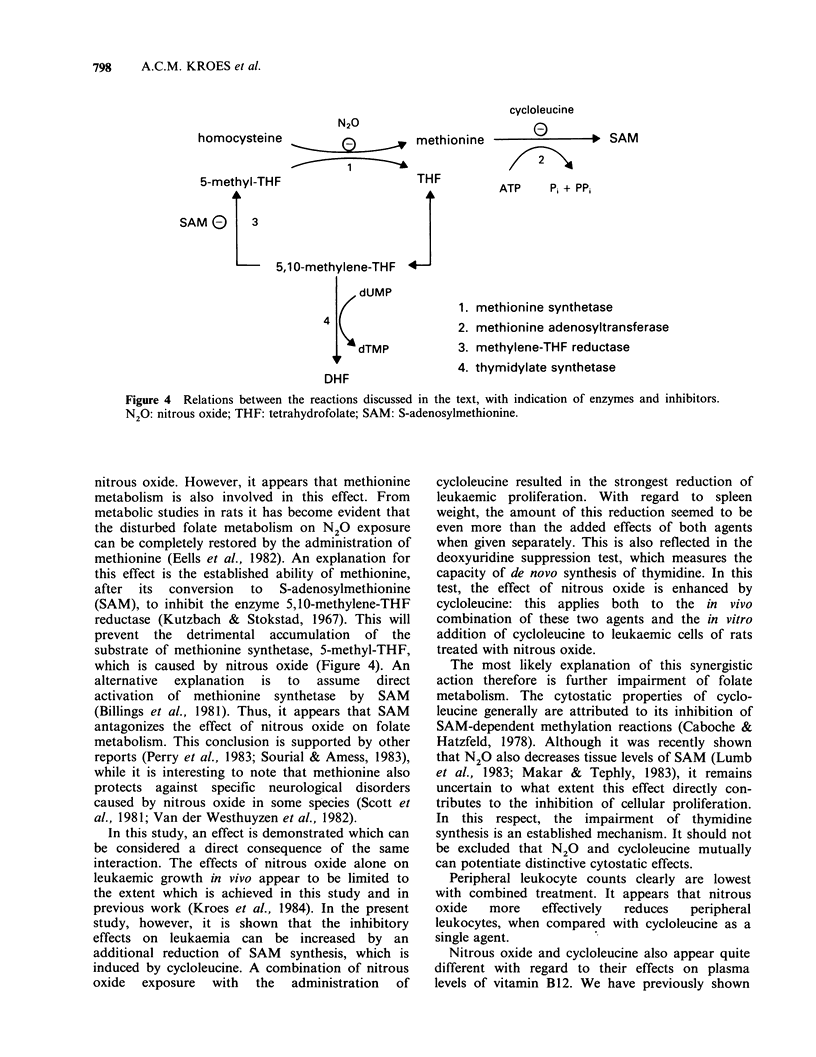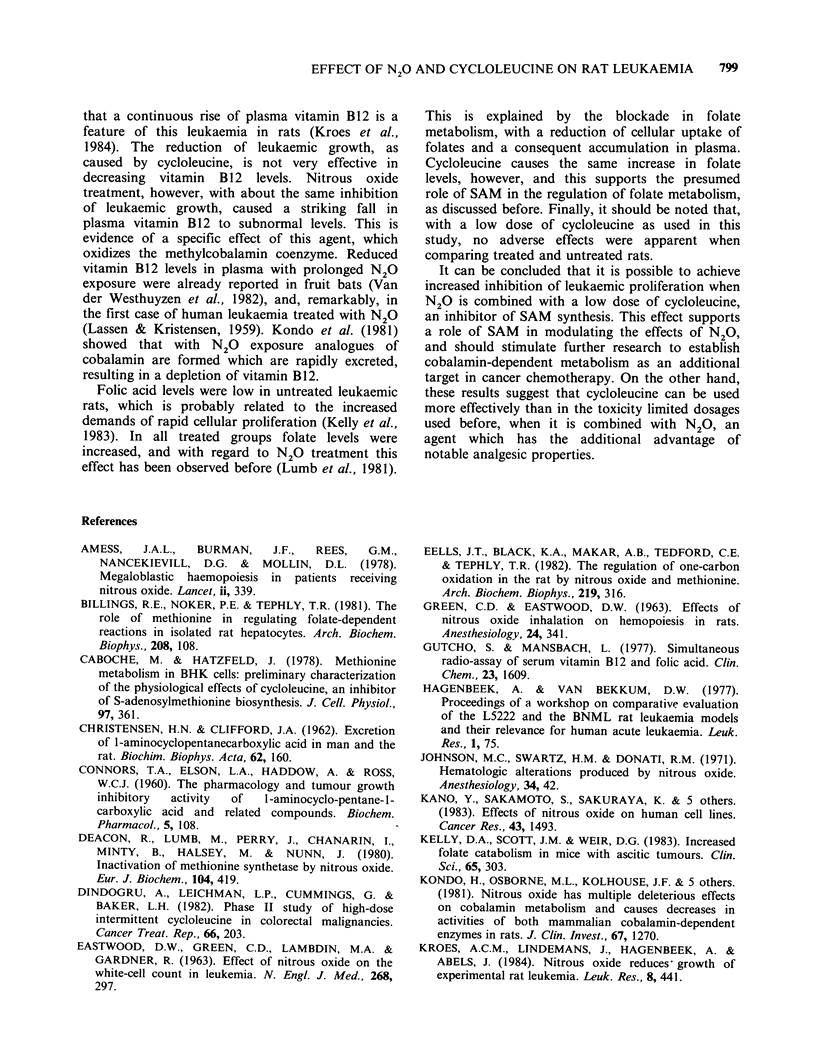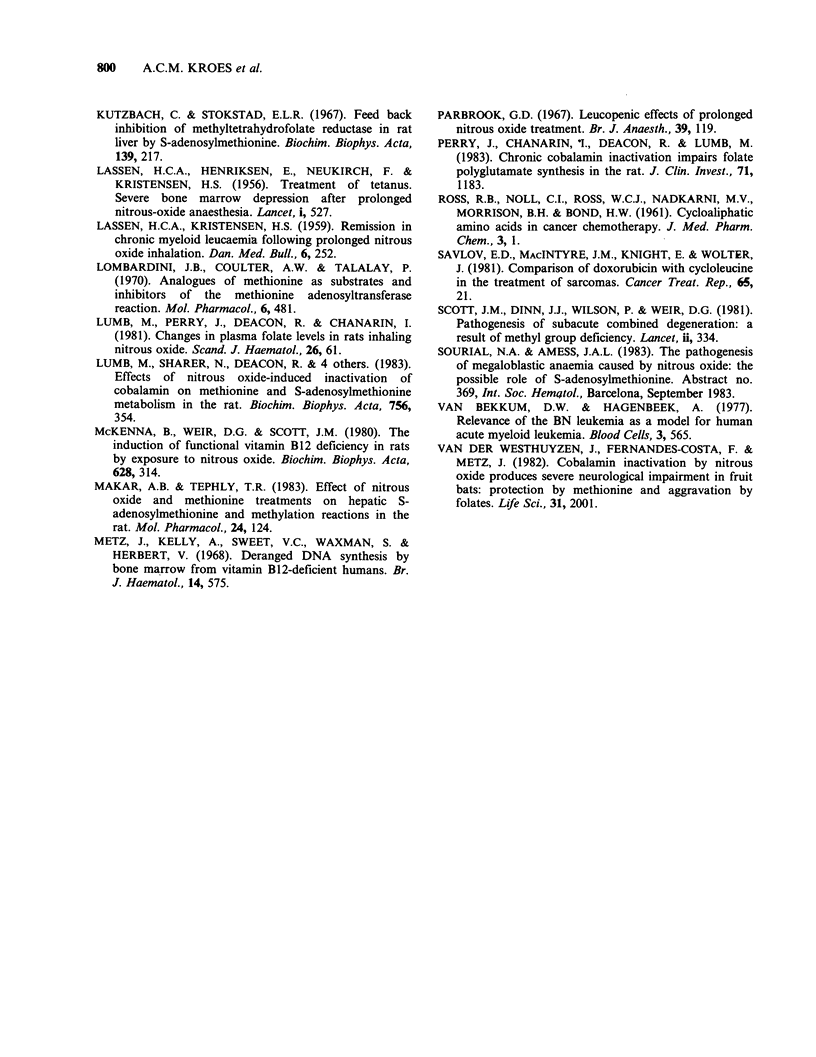# Synergistic growth inhibiting effect of nitrous oxide and cycloleucine in experimental rat leukaemia.

**DOI:** 10.1038/bjc.1984.258

**Published:** 1984-12

**Authors:** A. C. Kroes, J. Lindemans, J. Abels

## Abstract

Nitrous oxide (N2O) inactivates the vitamin B12-dependent enzyme methionine synthetase with subsequent impairment of folate metabolism and a reduction of cellular proliferation. Indications exist that this effect is antagonized by S-adenosylmethionine (SAM), and it was investigated whether combination with an inhibitor of SAM synthesis, cycloleucine, would result in increased inhibition of growth in rat leukaemia model (BNML). Leukaemic growth was compared in untreated rats, in rats treated with either nitrous oxide/oxygen (1:1) or cycloleucine (50 mg kg-1 i.p.), and in rats receiving both agents. Combined treatment resulted in the strongest reduction of leukaemic infiltration in spleen and liver, and this reduction often was more than the added effects of single treatments. Peripheral leukocyte counts were also lowest after combined treatment. The deoxyuridine suppression test, measuring folate-dependent de novo synthesis of thymidine, was more severely disturbed with combined treatment. Levels of vitamin B12 in plasma were reduced in rats receiving N2O, but an increase in plasma folate occurred in all treated rats. These results indicate that a reduction of SAM synthesis by cycloleucine can increase the disturbance of folate metabolism that is caused by nitrous oxide, with a potentiation of the effects on leukaemic growth.


*Br. J. Cancer* (1984), **50**, 793–800

### Synergistic growth inhibiting effect of nitrous oxide and cycloleucine in experimental rat leukaemia

A.C.M. Kroes, J. Lindemans & J. Abels

*Institute of Haematology, Erasmus University, P.O. Box 1738, 3000 DR Rotterdam, The Netherlands.*

**Summary** Nitrous oxide ($N_2O$) inactivates the vitamin B12-dependent enzyme methionine synthetase with subsequent impairment of folate metabolism and a reduction of cellular proliferation. Indications exist that this effect is antagonized by S-adenosylmethionine (SAM), and it was investigated whether combination with an inhibitor of SAM synthesis, cycloleucine, would result in increased inhibition of growth in rat leukaemia model (BNML). Leukaemic growth was compared in untreated rats, in rats treated with either nitrous oxide/oxygen (1:1) or cycloleucine ($50\,mg\,kg^{-1}$ i.p.), and in rats receiving both agents. Combined treatment resulted in the strongest reduction of leukaemic infiltration in spleen and liver, and this reduction often was more than the added effects of single treatments. Peripheral leukocyte counts were also lowest after combined treatment. The deoxyuridine suppression test, measuring folate-dependent *de novo* synthesis of thymidine, was more severely disturbed with combined treatment. Levels of vitamin B12 in plasma were reduced in rats receiving $N_2O$, but an increase in plasma folate occurred in all treated rats. These results indicate that a reduction of SAM synthesis by cycloleucine can increase the disturbance of folate metabolism that is caused by nitrous oxide, with a potentiation of the effects on leukaemic growth.

The well known anaesthetic gas nitrous oxide ($N_2O$) is able to suppress haematopoiesis, giving rise to megaloblastic anaemia in man (Lassen *et al.*, 1956) and marked leukopenia in rats (Green & Eastwood, 1963; Parbrook, 1967; Johnson *et al.*, 1971). This effect could be explained by a specific oxidative action of nitrous oxide on the cobalt-moiety of vitamin B12 (Amess *et al.*, 1978), which causes a nearly complete inactivation of the methyl-cobalamin-requiring enzyme methionine synthetase or 5-methyltetrahydrofolate homocysteine methyl-transferase (E.C. 2.1.1.13). This results in a decreased availability of tetrahydrofolate coenzymes, which impairs folate dependent *de novo* synthesis of thymidine and ultimately affects cellular proliferation. Attempts to utilize this haematological side effect of nitrous oxide in the treatment of human leukaemia were already reported before its biochemical mechanism was revealed (Lassen & Kristensen, 1959; Eastwood *et al.*, 1963). These preliminary trials showed some promising results with a rapid, though reversible, regression of leukaemia on nitrous oxide exposure. Recent investigations have confirmed inhibition of tumour growth *in vitro*, in several human cell lines (Kano *et al.*, 1983), and *in vivo*, in leukaemic rats (Kroes *et al.*, 1984).

The use of nitrous oxide in metabolic studies made clear that the effects of this agent, through a reduction of methionine supply, also involve a decreased synthesis of S-adenosylmethionine (SAM) (Lumb *et al.*, 1983; Makar & Tephly, 1983). In addition, it appeared that SAM antagonized the disturbance of folate metabolism which is caused by nitrous oxide (Eells *et al.*, 1982; Sourial & Amess, 1983). These observations suggest that a further reduction of SAM might enhance the inhibiting effects of nitrous oxide on cellular proliferation.

We now report the effect of combining nitrous oxide with cycloleucine (NSC-1026, 1-aminocyclo-pentane carboxylic acid). Cycloleucine is a potent inhibitor of the enzyme methionine adenosyl-transferase (E.C. 2.5.1.6.) (Lombardini *et al.*, 1970), which converts methionine into SAM. The combined action of nitrous oxide and cycloleucine can cause a sequential blockade of SAM synthesis, possibly resulting in increased inhibition of tumour growth. Interestingly, cycloleucine by itself also has cytostatic properties (Connors *et al.*, 1960; Ross *et al.*, 1961). However, in recent clinical trials this drug, in high doses, was unsuccessful mainly because of severe neurological and haematological toxicity (Savlov *et al.*, 1981; Dindogru *et al.*, 1982).

This study describes the effects of nitrous oxide exposure, combined with the administration of cycloleucine, on proliferation of an experimental rat leukaemia: the Brown Norway Myeloid Leukaemia (BNML). This transplantable leukaemia has been described in detail (Hagenbeek & Van Bekkum, 1977) and is considered to be a suitable model for experimental chemotherapy (Van Bekkum & Hagenbeek, 1977). To evaluate the metabolic effects of vitamin B12-inactivation by nitrous oxide, the deoxyuridine suppression test is

Correspondence: A.C.M. Kroes
Received 16 July 1984; accepted 29 September 1984.

© The Macmillan Press Ltd., 1984

used, and plasma levels are determined of vitamin B12 and folic acid.

### Materials and methods

### *Animals*

Male rats of the Brown Norway inbred strain were used, at the age of 14–18 weeks (body wt. 200–290 g). Food and water were supplied *ad libitum* during the experiments.

### *Brown Norway myelocytic leukaemia (BNML)*

Cryopreserved leukaemic cells were kindly provided by Dr A. Hagenbeek from the Radiobiological Institute TNO, Rijswijk, the Netherlands, where this transplantable rat leukaemia model was developed. Origin, classification and proliferation kinetics were described before (Hagenbeek & Van Bekkum, 1977). For leukaemia transfer in experimental series, spleen cells of fully leukaemic animals were used. $10^7$ cells, suspended in 1 ml of Hanks balanced salt solution were injected i.v. This standard dose leads to death in 20–24 days. During this period massive leukaemic infiltration of spleen, liver and bone marrow takes place. Spleen and liver weights, therefore, are reliable indicators of tumour load and, along with haematological determinations, can be used effectively to assess the effects of chemotherapy, correlating well with studies of survival time (Hagenbeek & Van Bekkum, 1977). To avoid a gradual change in growth properties, serial transplantations were limited to only two passages in order, after which spleen cells were used from animals of a separate series, freshly inoculated with cells from a cryopreserved stock.

### *Treatment with nitrous oxide and cycloleucine*

Two sets of identical experiments were carried out separately (Experiments 1 and 2). In each, 4 groups of 4 rats were inoculated with leukaemic cells at Day 0. Mean body weight in each group was nearly identical. Treatment of these leukaemic rats started at Day 7. To detect any synergistic action, one group received no treatment (controls), while the other 3 groups were treated with nitrous oxide, cycloleucine, or both. Rats were exposed to nitrous oxide in a 36 l flow chamber, in which a mixture of 50% nitrous oxide and 50% oxygen was blown at a rate of about 1000 ml min$^{-1}$. Oxygen concentration was monitored with an oxygen analyzer (Teledyne Analytical Instruments). Exposure was interrupted only for short cleaning periods. Rats not exposed to $N_2O$ were kept in air, but otherwise treated identically. Cycloleucine was administered as a single i.p. injection at Day 7, of 50 mg kg$^{-1}$ cycloleucine (Sigma Chemical Co., St Louis, USA), dissolved in water containing 0.15 mol l$^{-1}$ NaCl. Rats not receiving cycloleucine were injected with 0.15 mol l$^{-1}$ NaCl solution i.p.

### *Evaluation of treatment*

To allow a simultaneous investigation of several parameters of leukaemic growth, including metabolic tests, both experiments were terminated after a fixed period of 19 days of leukaemia, which is just before the rats would die spontaneously. Rats were killed by exsanguination under either anaesthesia, after recording their body weights. Liver and spleen were weighed. Leucocytes and thrombocytes were counted electronically. Haemoglobin concentration was measured by the haemoglobin cyanide spectrophotometrical assay. Plasma vitamin B12 and folic acid were determined simultaneously, essentially as described by Gutcho & Mansbach (1977). Methyltetrahydrofolate was used as a folate standard. Normal values for organ weights, haematological parameters, plasma folate and vitamin B12 concentrations were derived from at least 12 comparable non-leukaemic Brown Norway rats.

### *Deoxyuridine suppression test*

This test is used to evaluate the metabolic inactivation of vitamin B12 [$^3$H]-thymidine incorporation in DNA is measured with and without added deoxyuridine. Deoxyuridine is able to suppress incorporation of [$^3$H]-thymidine very significantly if it can be converted to thymidine by folate-dependent methylation. This suppression is decreased in bone marrow cells as a result of folate or vitamin B12 deficiency, and as a result of the inactivation of methylcobalamin by nitrous oxide (McKenna *et al.*, 1980).

In this test, leukaemic spleen cells were used ($\sim 5 \times 10^6$ per test) from rats of the various groups in Experiment 2, as described above. In addition, leukaemic rats were included who were similarly treated for 1 day only (Day 16–17), after which they were sacrificed. In some cases, cycloleucine was added to the leukaemic cell suspensions used in the test to compare *in vitro* and *in vivo* effects of this drug. The test was carried out according to Metz *et al.* (1968) with some modification as described before (Kroes *et al.*, 1984). Deoxyuridine (Sigma Chemical Co., St Louis, USA), was used in a concentration of 0.1 mmol l$^{-1}$. Incorporation of [$^3$H]-thymidine ($\sim 0.3\,\mu$Ci per test, specific activity 25 Ci mmol$^{-1}$, from Amersham International, UK), is expressed as a percentage of the maximum incorporation in each case, obtained by omitting deoxyuridine.

### Results

In each of the two experiments, four groups of leukaemic rats were compared: one untreated (controls), one treated with nitrous oxide, one treated with cycloleucine, and one group treated with both agents. Data on leukaemic growth in these groups can be found in Tables I and II. All rats survived until termination of the experiments, except for one untreated rat in Experiment 2, dying spontaneously a few hours before. Organ weights of this rat have been included in the results.

The treated rats had reduced spleen and liver weights (Table I) and reduced leukocyte counts (Table II), compared to the untreated controls. With Wilcoxon's non-parametric rank sum rest, applied to the values of individual rats in both experiments, these differences are statistically significant ($P<0.01$). Combined treatment resulted in the strongest inhibition of leukaemic growth, with an increase in organ weights of less than half of control values, as shown in Figure 1. The differences between groups treated with a single agent, and groups with combined treatment are also statistically significant using Wilcoxon's test ($P<0.01$).

No significant difference was observed between the two groups treated with nitrous oxide or cycloleucine alone. In fact, after preliminary experiments, the lowest dose of cycloleucine was selected which produced inhibition of growth about comparable to nitrous oxide exposure alone. This would facilitate recognition of a potentiating effect after combined treatment and minimize possible side effects of cycloleucine. With regard to the haematological values shown in Table II, platelet counts and haemoglobin values do not suggest adverse effects of treatment on normal haematopoiesis. Although all animals remained thrombopenic, haemoglobin values showed a small increase with treatment and were highest after combined treatment. These differences were, however, not statistically significant.

Treatment with $N_2O$, as in these experiments, is

**Table I** Effects of treatment on leukaemic infiltration in spleen and liver, and on body weight

| | *Spleen weight*[a] | | *Liver weight*[b] | | *Body weight* |
|---|---|---|---|---|---|
| *Treatment* | *Experiment 1* $g \pm s.e.$ | *Experiment 2* $g \pm s.e.$ | *Experiment 1* $g \pm s.e.$ | *Experiment 2* $g \pm s.e.$ | *Experiment 1+2* *% change* |
| None (controls) | 4.52±0.18 | 4.49±0.15 | 21.38±0.60 | 20.54±1.58 | −1.0 |
| Cycloleucine | 3.77±0.13 | 4.04±0.13 | 16.79±0.54 | 16.95±0.65 | +0.2 |
| Nitrous oxide | 3.57±0.14 | 3.34±0.23 | 18.81±0.30 | 15.50±0.93 | −5.9 |
| Cycloleucine and nitrous oxide | 2.32±0.08 | 2.42±0.05 | 13.04±0.54 | 13.48±0.51 | −4.1 |

All treatment groups in both experiments consisted of 4 rats.
s.e. = standard error of the mean in each group.
[a]normal spleen weight in comparable non-leukaemic Brown-Norway rats: 0.45±0.07 g.
[b]normal liver weight in comparable non-leukaemic Brown Norway rats: 8.25±0.99 g.

**Table II** Effects of treatment on haematological values

| | *Leukocytes*[a] | | *Thrombocytes*[b] | *Haemoglobin* |
|---|---|---|---|---|
| *Treatment* | *Experiment 1* $10^9 l^{-1} \pm s.e.$ | *Experiment 2* $10^9 l^{-1} \pm s.e.$ | *Experiment 1+2* $10^9 l^{-1} \pm s.e.$ | *Experiment 1+2* $mmol\, l^{-1} \pm s.e.$ |
| None (controls) | 32.7±2.3 | 19.2±1.2 | 81±13 | 8.0±0.2 |
| Cycloleucine | 30.3±3.9 | 11.8±0.6 | 94±13 | 8.7±0.1 |
| Nitrous oxide | 10.2±0.7 | 8.9±1.4 | 60± 5 | 8.2±0.3 |
| Cycloleucine and nitrous oxide | 5.3±0.3 | 5.2±0.6 | 61± 8 | 9.2±0.1 |

All treatment groups in both experiments consisted of 4 rats.
s.e. = standard error of the mean in each group.
[a]normal value of leukocyte count in Brown Norway rats: $3.7 \pm 0.3\ 10^9 l^{-1}$.
[b]normal value of thrombocyte count in Brown Norway rats: $790 \pm 22.10^9 l^{-1}$.

**Figure 1** The percentages of increase in weight are indicated of (a) spleen and (b) liver in leukaemic rats, relative to mean normal weights of spleen: 0.45 g and liver: 8.25 g in comparable non-leukaemic Brown Norway rats. Data of Experiments 1 and 2 are combined. Each group consists of 8 rats. S.e.m. are indicated.

well tolerated by rats, without a noticeable influence on consciousness. A limited loss of body weight is observed, which is not aggravated by cycloleucine (Table I).

Plasma concentrations of vitamin B12 and folic acid as determined in the various groups of both experiments, are shown in Figure 2. In untreated leukaemic rats a strong increase in plasma levels of vitamin B12 is found, whereas decreases are observed after treatment. A pronounced effect of nitrous oxide is evident, in particular when compared with the effect of cycloleucine. Combined treatment resulted in the lowest levels of vitamin B12. Folic acid levels are low in untreated rats, with treatment resulting in higher levels. In this case, no differences are observed between the various treatments.

Results of deoxyuridine suppression tests are presented in Figure 3. In this test, higher values are caused by a reduced suppressive effect of deoxyuridine on the incorporation of [$^3$H]-thymidine, which is indicative of impaired *de novo* synthesis of thymidine. Figure 3(a) shows suppression values, obtained with leukaemic spleen cells from rats of the four groups in Experiment 2. Although the differences are small, they are suggestive of a limited increase in suppression values after $N_2O$ or cycloleucine as single treatments, with a more severe disturbance after combined treatment. These values, however, were obtained 12 days after the initiation of treatment in Experiment 2. In this period, the level of cycloleucine may be appreciably reduced, although this agent in normal rats has a plasma half life of about 22 days (Christensen & Clifford, 1962). Therefore, in a separate experiment leukaemic spleen cells were used of rats 1 day after the administration of cycloleucine (Figure 3b). This resulted in a more pronounced effect after combined treatment. About the same values were observed with *in vitro* addition of cycloleucine to leukaemic cell suspensions of untreated rats and rats treated with $N_2O$. At a concentration of 1 mmol $l^{-1}$, effects on the deoxyuridine suppression test are comparable with *in vivo* administration of 50 mg $kg^{-1}$ cycloleucine.

### Discussion

Exposure to $N_2O$ is known to cause a selective and virtually complete inhibition of the methyl-cobalamin-dependent enzyme methionine synthetase (Deacon *et al.*, 1980). This enzyme is essential both for the generation of tetrahydrofolate (THF) and the synthesis of methionine (see Figure 4). The disturbance of folate metabolism, and in particular of folate-dependent *de novo* synthesis of thymidine, is considered to be primarily responsible for the impairment of cellular proliferation caused by

**Figure 2** Plasma levels of (a) vitamin B12 and (b) folic acid in leukaemic rats of Experiments 1 and 2. Values in normal (non-leukaemic) rats are also shown. S.e.m. are indicated.

**Figure 3** Deoxyuridine suppression values in leukaemic cells: (a) from four groups of two rats each, 12 days after the administration of cycloleucine and initiation of nitrous oxide exposure; (b) from four rats, after treatment for 1 day, with cycloleucine administered *in vivo* (columns 2 and 4), and cycloleucine added *in vitro* to leukaemic cell suspensions (columns 5 and 6). Each value is the mean of triple incubations, with a maximal difference between estimations of 10%. dU: deoxyuridine.

**Figure 4** Relations between the reactions discussed in the text, with indication of enzymes and inhibitors. $N_2O$: nitrous oxide; THF: tetrahydrofolate; SAM: S-adenosylmethionine.

nitrous oxide. However, it appears that methionine metabolism is also involved in this effect. From metabolic studies in rats it has become evident that the disturbed folate metabolism on $N_2O$ exposure can be completely restored by the administration of methionine (Eells *et al.*, 1982). An explanation for this effect is the established ability of methionine, after its conversion to S-adenosylmethionine (SAM), to inhibit the enzyme 5,10-methylene-THF reductase (Kutzbach & Stokstad, 1967). This will prevent the detrimental accumulation of the substrate of methionine synthetase, 5-methyl-THF, which is caused by nitrous oxide (Figure 4). An alternative explanation is to assume direct activation of methionine synthetase by SAM (Billings *et al.*, 1981). Thus, it appears that SAM antagonizes the effect of nitrous oxide on folate metabolism. This conclusion is supported by other reports (Perry *et al.*, 1983; Sourial & Amess, 1983), while it is interesting to note that methionine also protects against specific neurological disorders caused by nitrous oxide in some species (Scott *et al.*, 1981; Van der Westhuyzen *et al.*, 1982).

In this study, an effect is demonstrated which can be considered a direct consequence of the same interaction. The effects of nitrous oxide alone on leukaemic growth *in vivo* appear to be limited to the extent which is achieved in this study and in previous work (Kroes *et al.*, 1984). In the present study, however, it is shown that the inhibitory effects on leukaemia can be increased by an additional reduction of SAM synthesis, which is induced by cycloleucine. A combination of nitrous oxide exposure with the administration of cycloleucine resulted in the strongest reduction of leukaemic proliferation. With regard to spleen weight, the amount of this reduction seemed to be even more than the added effects of both agents when given separately. This is also reflected in the deoxyuridine suppression test, which measures the capacity of *de novo* synthesis of thymidine. In this test, the effect of nitrous oxide is enhanced by cycloleucine: this applies both to the *in vivo* combination of these two agents and the *in vitro* addition of cycloleucine to leukaemic cells of rats treated with nitrous oxide.

The most likely explanation of this synergistic action therefore is further impairment of folate metabolism. The cytostatic properties of cycloleucine generally are attributed to its inhibition of SAM-dependent methylation reactions (Caboche & Hatzfeld, 1978). Although it was recently shown that $N_2O$ also decreases tissue levels of SAM (Lumb *et al.*, 1983; Makar & Tephly, 1983), it remains uncertain to what extent this effect directly contributes to the inhibition of cellular proliferation. In this respect, the impairment of thymidine synthesis is an established mechanism. It should not be excluded that $N_2O$ and cycloleucine mutually can potentiate distinctive cytostatic effects.

Peripheral leukocyte counts clearly are lowest with combined treatment. It appears that nitrous oxide more effectively reduces peripheral leukocytes, when compared with cycloleucine as a single agent.

Nitrous oxide and cycloleucine also appear quite different with regard to their effects on plasma levels of vitamin B12. We have previously shown that a continuous rise of plasma vitamin B12 is a feature of this leukaemia in rats (Kroes *et al.*, 1984). The reduction of leukaemic growth, as caused by cycloleucine, is not very effective in decreasing vitamin B12 levels. Nitrous oxide treatment, however, with about the same inhibition of leukaemic growth, caused a striking fall in plasma vitamin B12 to subnormal levels. This is evidence of a specific effect of this agent, which oxidizes the methylcobalamin coenzyme. Reduced vitamin B12 levels in plasma with prolonged $N_2O$ exposure were already reported in fruit bats (Van der Westhuyzen *et al.*, 1982), and, remarkably, in the first case of human leukaemia treated with $N_2O$ (Lassen & Kristensen, 1959). Kondo *et al.* (1981) showed that with $N_2O$ exposure analogues of cobalamin are formed which are rapidly excreted, resulting in a depletion of vitamin B12.

Folic acid levels were low in untreated leukaemic rats, which is probably related to the increased demands of rapid cellular proliferation (Kelly *et al.*, 1983). In all treated groups folate levels were increased, and with regard to $N_2O$ treatment this effect has been observed before (Lumb *et al.*, 1981). This is explained by the blockade in folate metabolism, with a reduction of cellular uptake of folates and a consequent accumulation in plasma. Cycloleucine causes the same increase in folate levels, however, and this supports the presumed role of SAM in the regulation of folate metabolism, as discussed before. Finally, it should be noted that, with a low dose of cycloleucine as used in this study, no adverse effects were apparent when comparing treated and untreated rats.

It can be concluded that it is possible to achieve increased inhibition of leukaemic proliferation when $N_2O$ is combined with a low dose of cycloleucine, an inhibitor of SAM synthesis. This effect supports a role of SAM in modulating the effects of $N_2O$, and should stimulate further research to establish cobalamin-dependent metabolism as an additional target in cancer chemotherapy. On the other hand, these results suggest that cycloleucine can be used more effectively than in the toxicity limited dosages used before, when it is combined with $N_2O$, an agent which has the additional advantage of notable analgesic properties.

### References

AMESS, J.A.L., BURMAN, J.F., REES, G.M., NANCEKIEVILL, D.G. & MOLLIN, D.L. (1978). Megaloblastic haemopoiesis in patients receiving nitrous oxide. *Lancet*, **ii**, 339.

BILLINGS, R.E., NOKER, P.E. & TEPHLY, T.R. (1981). The role of methionine in regulating folate-dependent reactions in isolated rat hepatocytes. *Arch. Biochem. Biophys.*, **208**, 108.

CABOCHE, M. & HATZFELD, J. (1978). Methionine metabolism in BHK cells: preliminary characterization of the physiological effects of cycloleucine, an inhibitor of S-adenosylmethionine biosynthesis. *J. Cell. Physiol.*, **97**, 361.

CHRISTENSEN, H.N. & CLIFFORD, J.A. (1962). Excretion of 1-aminocyclopentanecarboxylic acid in man and the rat. *Biochim. Biophys. Acta*, **62**, 160.

CONNORS, T.A., ELSON, L.A., HADDOW, A. & ROSS, W.C.J. (1960). The pharmacology and tumour growth inhibitory activity of 1-aminocyclo-pentane-1-carboxylic acid and related compounds. *Biochem. Pharmacol.*, **5**, 108.

DEACON, R., LUMB, M., PERRY, J., CHANARIN, I., MINTY, B., HALSEY, M. & NUNN, J. (1980). Inactivation of methionine synthetase by nitrous oxide. *Eur. J. Biochem.*, **104**, 419.

DINDOGRU, A., LEICHMAN, L.P., CUMMINGS, G. & BAKER, L.H. (1982). Phase II study of high-dose intermittent cycloleucine in colorectal malignancies. *Cancer Treat. Rep.*, **66**, 203.

EASTWOOD, D.W., GREEN, C.D., LAMBDIN, M.A. & GARDNER, R. (1963). Effect of nitrous oxide on the white-cell count in leukemia. *N. Engl. J. Med.*, **268**, 297.

EELLS, J.T., BLACK, K.A., MAKAR, A.B., TEDFORD, C.E. & TEPHLY, T.R. (1982). The regulation of one-carbon oxidation in the rat by nitrous oxide and methionine. *Arch. Biochem. Biophys.*, **219**, 316.

GREEN, C.D. & EASTWOOD, D.W. (1963). Effects of nitrous oxide inhalation on hemopoiesis in rats. *Anesthesiology*, **24**, 341.

GUTCHO, S. & MANSBACH, L. (1977). Simultaneous radio-assay of serum vitamin B12 and folic acid. *Clin. Chem.*, **23**, 1609.

HAGENBEEK, A. & VAN BEKKUM, D.W. (1977). Proceedings of a workshop on comparative evaluation of the L5222 and the BNML rat leukaemia models and their relevance for human acute leukaemia. *Leuk. Res.*, **1**, 75.

JOHNSON, M.C., SWARTZ, H.M. & DONATI, R.M. (1971). Hematologic alterations produced by nitrous oxide. *Anesthesiology*, **34**, 42.

KANO, Y., SAKAMOTO, S., SAKURAYA, K. & 5 others. (1983). Effects of nitrous oxide on human cell lines. *Cancer Res.*, **43**, 1493.

KELLY, D.A., SCOTT, J.M. & WEIR, D.G. (1983). Increased folate catabolism in mice with ascitic tumours. *Clin. Sci.*, **65**, 303.

KONDO, H., OSBORNE, M.L., KOLHOUSE, J.F. & 5 others. (1981). Nitrous oxide has multiple deleterious effects on cobalamin metabolism and causes decreases in activities of both mammalian cobalamin-dependent enzymes in rats. *J. Clin. Invest.*, **67**, 1270.

KROES, A.C.M., LINDEMANS, J., HAGENBEEK, A. & ABELS, J. (1984). Nitrous oxide reduces growth of experimental rat leukemia. *Leuk. Res.*, **8**, 441.

KUTZBACH, C. & STOKSTAD, E.L.R. (1967). Feed back inhibition of methyltetrahydrofolate reductase in rat liver by S-adenosylmethionine. *Biochim. Biophys. Acta,* **139,** 217.

LASSEN, H.C.A., HENRIKSEN, E., NEUKIRCH, F. & KRISTENSEN, H.S. (1956). Treatment of tetanus. Severe bone marrow depression after prolonged nitrous-oxide anaesthesia. *Lancet,* **i,** 527.

LASSEN, H.C.A., KRISTENSEN, H.S. (1959). Remission in chronic myeloid leucaemia following prolonged nitrous oxide inhalation. *Dan. Med. Bull.,* **6,** 252.

LOMBARDINI, J.B., COULTER, A.W. & TALALAY, P. (1970). Analogues of methionine as substrates and inhibitors of the methionine adenosyltransferase reaction. *Mol. Pharmacol.,* **6,** 481.

LUMB, M., PERRY, J., DEACON, R. & CHANARIN, I. (1981). Changes in plasma folate levels in rats inhaling nitrous oxide. *Scand. J. Haematol.,* **26,** 61.

LUMB, M., SHARER, N., DEACON, R. & 4 others. (1983). Effects of nitrous oxide-induced inactivation of cobalamin on methionine and S-adenosylmethionine metabolism in the rat. *Biochim. Biophys. Acta,* **756,** 354.

McKENNA, B., WEIR, D.G. & SCOTT, J.M. (1980). The induction of functional vitamin B12 deficiency in rats by exposure to nitrous oxide. *Biochim. Biophys. Acta,* **628,** 314.

MAKAR, A.B. & TEPHLY, T.R. (1983). Effect of nitrous oxide and methionine treatments on hepatic S-adenosylmethionine and methylation reactions in the rat. *Mol. Pharmacol.,* **24,** 124.

METZ, J., KELLY, A., SWEET, V.C., WAXMAN, S. & HERBERT, V. (1968). Deranged DNA synthesis by bone marrow from vitamin B12-deficient humans. *Br. J. Haematol.,* **14,** 575.

PARBROOK, G.D. (1967). Leucopenic effects of prolonged nitrous oxide treatment. *Br. J. Anaesth.,* **39,** 119.

PERRY, J., CHANARIN, I., DEACON, R. & LUMB, M. (1983). Chronic cobalamin inactivation impairs folate polyglutamate synthesis in the rat. *J. Clin. Invest.,* **71,** 1183.

ROSS, R.B., NOLL, C.I., ROSS, W.C.J., NADKARNI, M.V., MORRISON, B.H. & BOND, H.W. (1961). Cycloaliphatic amino acids in cancer chemotherapy. *J. Med. Pharm. Chem.,* **3,** 1.

SAVLOV, E.D., MacINTYRE, J.M., KNIGHT, E. & WOLTER, J. (1981). Comparison of doxorubicin with cycloleucine in the treatment of sarcomas. *Cancer Treat. Rep.,* **65,** 21.

SCOTT, J.M., DINN, J.J., WILSON, P. & WEIR, D.G. (1981). Pathogenesis of subacute combined degeneration: a result of methyl group deficiency. *Lancet,* **ii,** 334.

SOURIAL, N.A. & AMESS, J.A.L. (1983). The pathogenesis of megaloblastic anaemia caused by nitrous oxide: the possible role of S-adenosylmethionine. Abstract no. 369, *Int. Soc. Hematol.,* Barcelona, September 1983.

VAN BEKKUM, D.W. & HAGENBEEK, A. (1977). Relevance of the BN leukemia as a model for human acute myeloid leukemia. *Blood Cells,* **3,** 565.

VAN DER WESTHUYZEN, J., FERNANDES-COSTA, F. & METZ, J. (1982). Cobalamin inactivation by nitrous oxide produces severe neurological impairment in fruit bats: protection by methionine and aggravation by folates. *Life Sci.,* **31,** 2001.